# The Rocky Mountain Interstate Medical Association

**Published:** 1897-08

**Authors:** E. Stuver

**Affiliations:** Secretary


### Correspondence.

### The Rocky Mountain Interstate Medical Association.

*Editor Texas Medical Journal:*

This is an organization of the regular medical profession of Colorado, Utah, Montana, Idaho, Wyoming, New Mexico and Arizona, for the purpose of promoting the scientific advancement, elevation and general welfare of the medical profession of these states and territories.

The idea of this society originated with Drs. C. K. Fleming, J. N. Hall, Leonard Freeman and Robert Levy of Denver, Colo., in the summer of 1896. A circular letter was sent by them to prominent physicians in the above mentioned states and territories, inviting their opinion as to the desirability of such a society, and their co-operation in its organization.

The plan received the hearty and almost universal approval of individual physicians, as well as the endorsement of the Colorado, Utah and Montana State Medical Societies. Accordingly a committee of three from each of the seven states was appointed to draft a constitution and by-laws, and to organize the society. This committee met at the Knutsford hotel, Salt Lake City, July 24, 1897, with the following members present, viz:

Colorado—Drs. C. K. Fleming, Clayton Parkhill and D. H. Coover, all of Denver.

Utah—Drs. C. P. Hough, C. G. Plummer and A. S. Bower, all of Salt Lake City.

Montana—Drs. C. K. Coe, of Helena; Henry Chapple, of Billings, and T. J. Murray, of Butte.

Wyoming—Dr. E. Stuver, of Rawlins.

The committee organized by electing C. P. Hough president, and E. Stuver secretary, and at once proceeded to the consideration and adoption of a constitution and by-laws. This work was very carefully and thoroughly done, and the Code of Ethics of the American Medical Association was adopted as the ethical guide of the society. This work being completed, the following officers were elected for the ensuing year, viz:

C. P. Hough, president, Salt Lake City, Utah; C. K. Cole, first vice-president, Helena, Mont., Clayton Parkhill, second vice-president, Denver, Colo.; E. Stuver, secretary and treasurer, Rawlins, Wyo. Committee on Membership—Drs. Henry Chapple, C. C. Fleming, A. S. Bower and C. G. Plummer.

It was decided to hold the first annual meeting in Denver, in June, 1898, during the meeting of the American Medical Association.

E. Stuver, Secretary.

